# Obstructive sleep apnea: A sharp increase in the prevalence of patients treated with nasal CPAP over the last decade in France

**DOI:** 10.1371/journal.pone.0245392

**Published:** 2021-01-12

**Authors:** Laurence Mandereau-Bruno, Damien Léger, Marie-Christine Delmas

**Affiliations:** 1 Santé Publique France, The French National Public Health Agency, Saint-Maurice, France; 2 EA 7330 VIFASOM and APHP-Hôtel Dieu, Centre du Sommeil et de la Vigilance, Université de Paris, Paris, France; Harper University Hospital, UNITED STATES

## Abstract

**Introduction:**

Obstructive sleep apnea (OSA) is a frequent condition. In the absence of treatment, OSA is associated with a higher risk of traffic accidents and a large variety of diseases. The objectives of this study were to describe the characteristics of patients treated for OSA in France and assess the time trends in treatment.

**Methods:**

The French National Health Data System is an individual database with data on all healthcare reimbursements for the entire French population. Based on this database, we included all patients aged 20 years or over who were treated with continuous positive airway pressure (CPAP) or mandibular advancement splint (MAS) between 2009 and 2018. Negative binomial models, adjusted for age, were used to assess time trends in treatment prevalence and incidence rates.

**Results:**

In 2017, 2.3% of French adults aged ≥20 years were treated with CPAP (men: 3.3%; women: 1.3%). The highest prevalence was observed in people aged 70–74 years (5.0%). From 2009 to 2018, the annual prevalence of CPAP increased 3-fold and the annual incidence 1.9-fold. During the same period, the rate of patients reimbursed for MAS (first prescription or renewal) was multiplied by 7.6. The proportion of patients treated with CPAP in 2017 who were no longer treated in the subsequent year was 6.9%.

**Discussion:**

The sharp increase in the incidence of OSA treatment probably reflects a better recognition of the disease in France. However, the prevalence of OSA treatment remains lower than expected based on the international literature. Further studies are needed to identify the obstacles to an optimal management of individuals with OSA in France.

## Introduction

It is well known that obstructive sleep apnea (OSA) may interfere with numerous metabolic and cardiovascular risks [1]. OSA is also associated with a higher risk of road accidents due to daytime sleepiness as well as cognitive disorders. OSA is defined as a combination of clinical symptoms, dominated by excessive daytime sleepiness not explained by other factors, a feeling of non-restorative sleep, and at least five apneas or hypopneas per hour of sleep [2].

The estimated prevalence of OSA varies greatly from one study to another due to differences in the criteria used to define it (method of diagnosis, apnea/hypopnea threshold, presence of symptoms) as well as variations in the populations studied, especially in terms of body mass index (BMI) and age, which are major risk factors for OSA. On the basis of a single threshold of five apneas/hypopneas per hour, studies observe rates in adults ranging from 9% to 38% [3]. Although an awareness of OSA is growing among health professionals and the general population, it remains underdiagnosed. There is also a reticence among many patients and health professionals to accept the recommended treatment.

The treatment of OSA is adapted to the symptoms and severity of the syndrome, measured by the apnea-hypopnea index (AHI) [4]. In France, the Health Authority recommends treating patients with moderate or severe OSA defined as AHI≥15/hr and at least three of the following symptoms: daytime sleepiness, habitual loud snoring, feeling of choking or suffocation during sleep, daytime fatigue, nocturia, and morning headaches. The choice of medical device–nocturnal continuous positive airway pressure (CPAP) or mandibular advancement splint (MAS)–depends on the severity of symptoms. CPAP is recommended as first-line treatment when the AHI exceeds 30/hr, or when the AHI is between 15 and 30/hr and is associated with poor-quality sleep (at least 10 microarousals per hour of sleep) or concomitant severe cardiovascular disease. In all circumstances, MAS is an alternative if CPAP is refused or poorly tolerated by the patient. MAS is recommended as first-line treatment when the AHI is between 15 and 30 and there is no associated severe cardiovascular disease. In all cases, irrespective of OSA severity, lifestyle and dietary advice is offered, irrespective of OSA severity.

The purpose of our analysis was to describe the characteristics of patients treated for OSA in France and to assess the time trends in the prevalence and incidence of OSA treatment over the last decade.

## Methods

### Source of data

Data are drawn from the French National Health Data System (SNDS) [5]. The SNDS comprises a database of anonymized individual data known as the DCIR, which includes all reimbursements for care delivered on an outpatient basis (medical acts, laboratory tests, medical devices, medicinal products) as well as social and demographic data (age, sex, entitlement to complementary universal health insurance (CMU-C), area of residence, date of death) for the recipients of these services and information about the health professionals consulted. Medical diagnoses are not stipulated unless they are provided as the reason for exemption from a co-payment relating to a long-term condition (affection de longue durée, ALD), an occupational illness, a workplace accident, or a disability. To date, the DCIR collects data from all health insurance schemes except for the National Assembly and Senate schemes. CPAP devices and, since late 2008, MAS are on the list of reimbursable products and services. For a CPAP device to be covered by the patient’s health insurance in France, a prior agreement is required based on the patient’s clinical and polygraphic/polysomnographic evaluation: i) AHI≥30/hr; or ii) if AHI≥15 and <30, then severe daytime sleepiness, accident risk, or severe cardiovascular or respiratory comorbidity. Hypopnea is defined as at least a 30% reduction in airflow amplitude lasting for at least 10 seconds and associated with a 3% or more oxygen desaturation. This agreement for a CPAP device must be renewed every year. MAS renewal is covered every 2 years.

### Study population

The analysis focused on health insurance beneficiaries aged 20 or older and residing in France who received at least one reimbursement for the use of a CPAP or MAS between 2009 and 2018. We examined all the codes for reimbursable products and services relating to CPAP, irrespective of whether CPAP use was associated with oxygen therapy, as well as all the codes relating to MAS.

### Data analysis

A patient was considered to have been treated with CPAP in a given year if CPAP treatment was delivered during the year (prevalent case), and as newly treated (incident case) if CPAP treatment was delivered in a given year but not in the previous year. After excluding patients who deceased in years N or N+1, patients treated in year N who did not receive treatment in the following year were considered to have stopped treatment. Regarding MAS, the annual numbers of reimbursed patients, including both patients who were newly treated and MAS renewals, were calculated.

The annual rates of prevalence and incidence of CPAP and MAS treatment were calculated using population data provided by the French National Institute for Statistics and Economic Studies (INSEE). For the analysis of socioeconomic disparities (based on the entitlement to CMU-C and the French deprivation index of the area of residence (FDep) [6], the annual number of patients was divided by the population of health insurance beneficiaries who received at least one reimbursement for care in the year under consideration. The FDep covers mainland France and only applies to beneficiaries of the general health insurance scheme, the scheme for self-employed workers (RSI), and the scheme for agricultural workers (MSA), representing around 85% of the population covered by health insurance.

To eliminate the effect of age in the comparisons, age-standardized annual prevalence and incidence rates were calculated using the European population as a reference for the age structure [7]. Time trends from 2009 to 2018 were assessed after excluding four health insurance schemes (accounting for less than 2% of health insurance beneficiaries) that were incorporated into the SNDS after 2009. The average annual percent changes were estimated using negative binomial regression models adjusted for age and sex. Interaction terms were added to the models to test for differences in time trends by sex or age. The analysis was performed using SAS software (SAS Institute Inc., Cary, NC, USA).

## Results

### Prevalence of CPAP treatment

**Fig 1:**
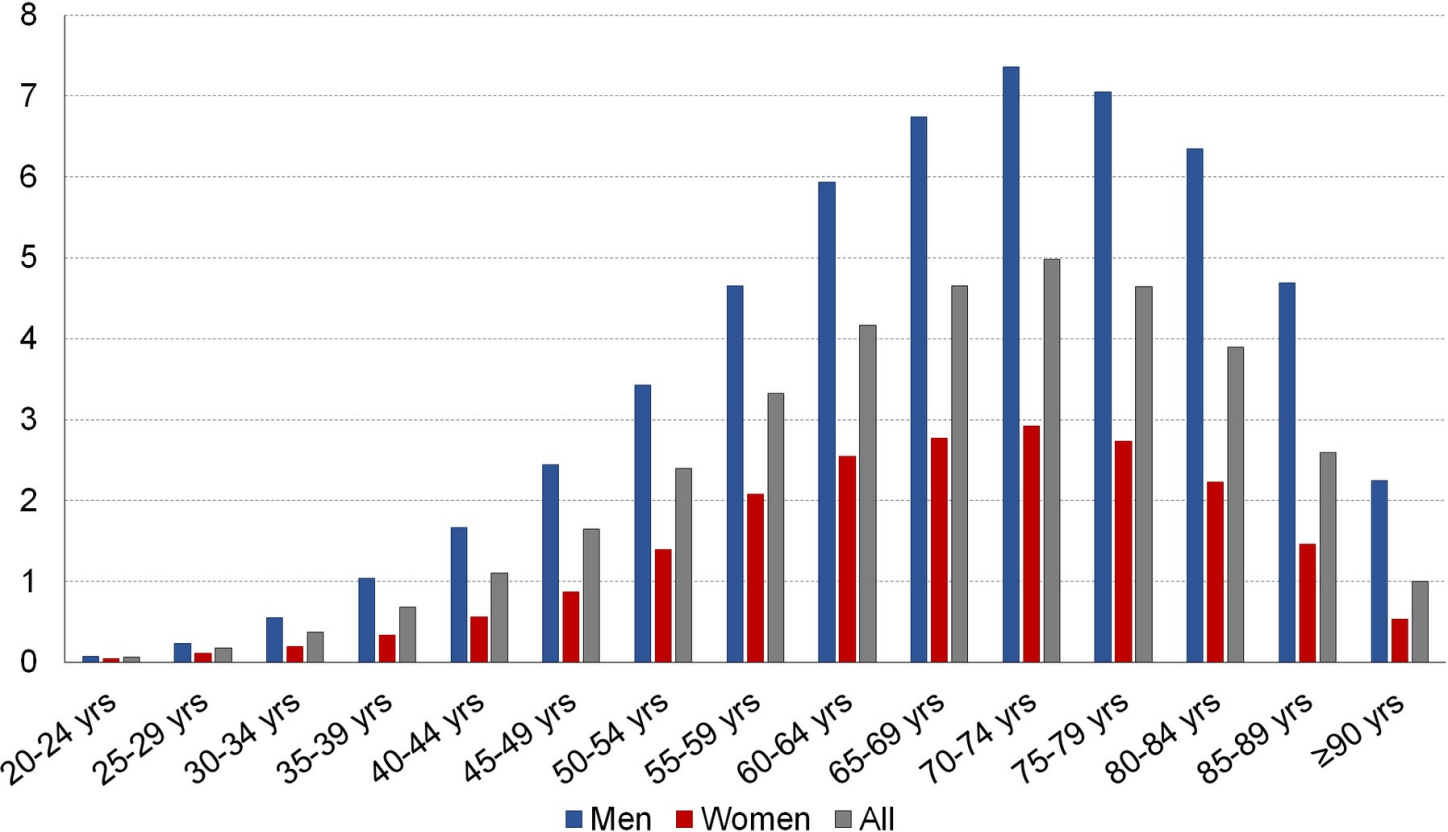
Prevalence (%) of treatment with continuous positive airway pressure by gender and age, France, 2017.

In 2017, 1,152,539 patients aged 20 years or older received treatment with CPAP, which corresponds to an annual prevalence of 2.3% (3.3% in men, 1.3% in women). The mean age of patients was 63 years. For all age ranges, prevalence was higher in men than in women (Fig 1). The age-specific rate increased regularly with age to reach a maximum in the age range of 70–74 years (5.0% for both sexes combined, 7.4% in men, 2.9% in women). In patients treated with CPAP in 2017 who belonged to the general health insurance scheme, 22.5% had a diagnosis of diabetes, 9.4% coronary heart disease, 8.4% another heart disease, and 2.5% a stroke-related disability (all ages combined).

**Fig 2:**
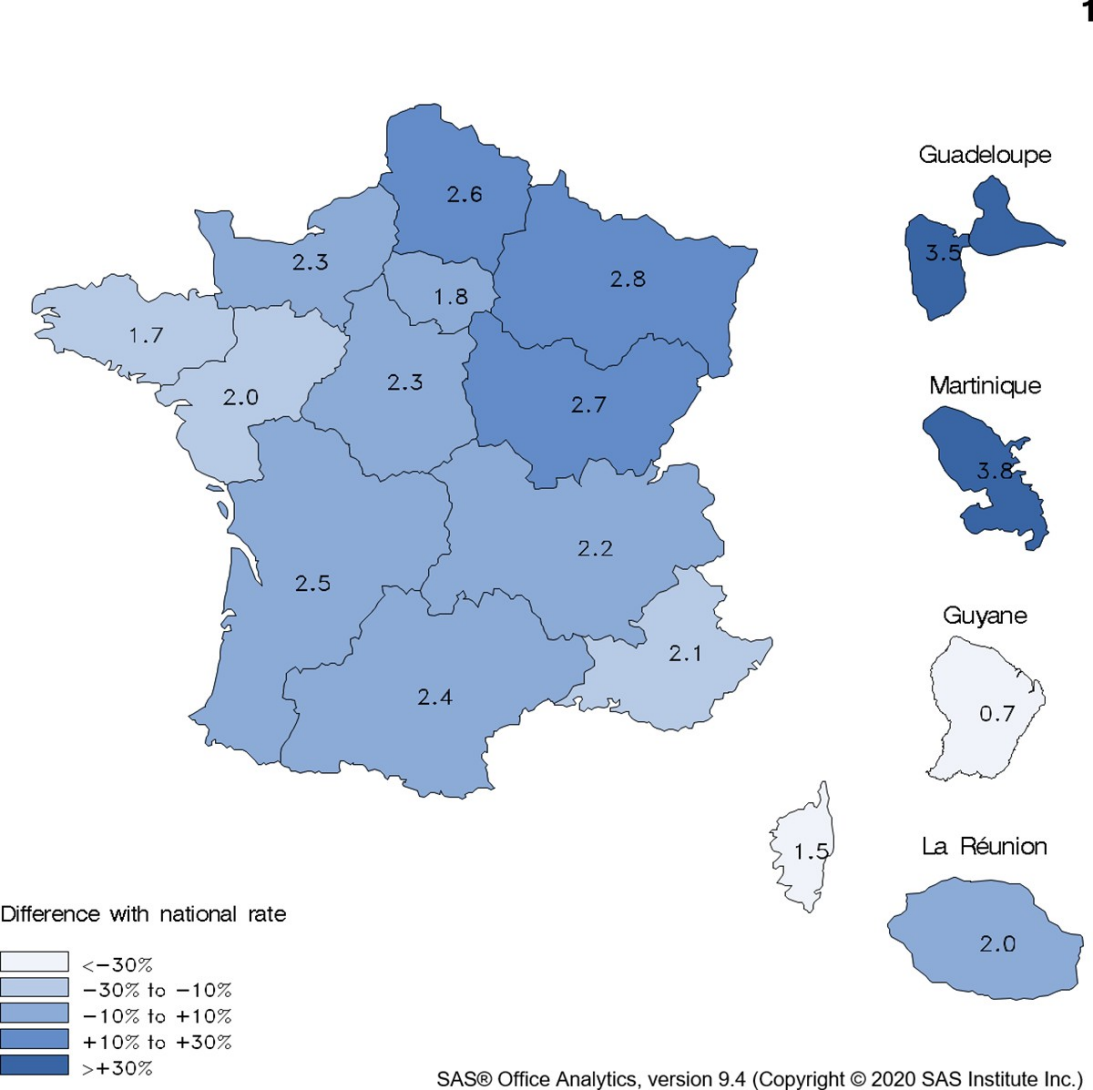
Crude prevalence (%) of treatment *with continuous positive airway pressure by region*, *and relative difference between the regional and national age-standardized rate*, *age ≥20 years*, *France*, *2017*.

Significant variations in the prevalence of CPAP treatment were observed in different French regions (Fig 2). The highest rates, after standardization for age, were in Martinique and Guadeloupe followed by the North and North-East in mainland France.

**Fig 3:**
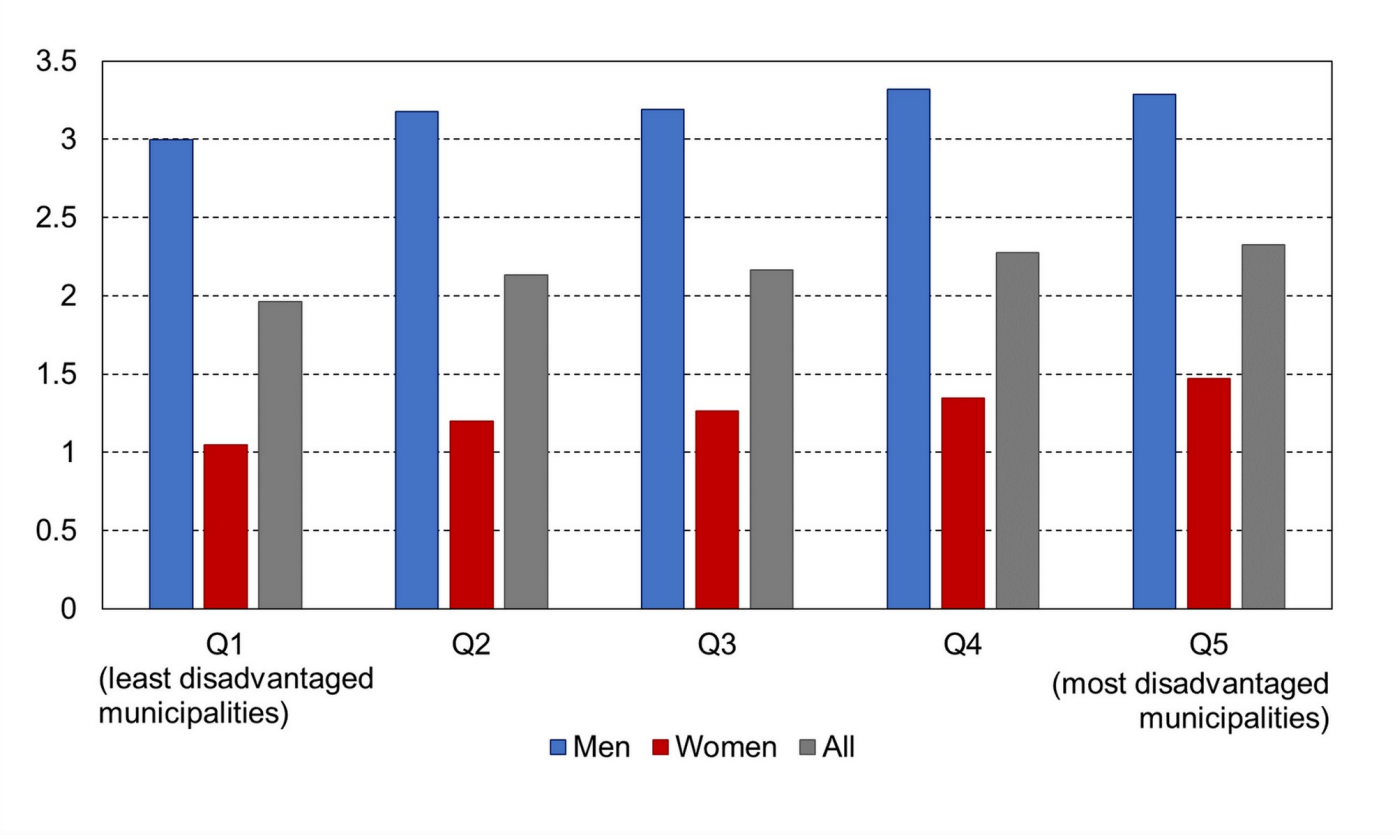
Prevalence (%) of treatment with continuous positive airway pressure according to the French deprivation index (by quintile) in people age ≥20 years, age-standardized rates, mainland France, 2017.

Among health insurance beneficiaries aged between 20 and 59 years, the standardized prevalence of CPAP treatment was similar for those with and without CMU-C (1.3% for both). In mainland France, the differences based on the deprivation index (FDep) were more marked in women than in men (the prevalence ratio of the most disadvantaged quintile to the least was 1.40 in women and 1.10 in men) (Fig 3).

**Fig 4:**
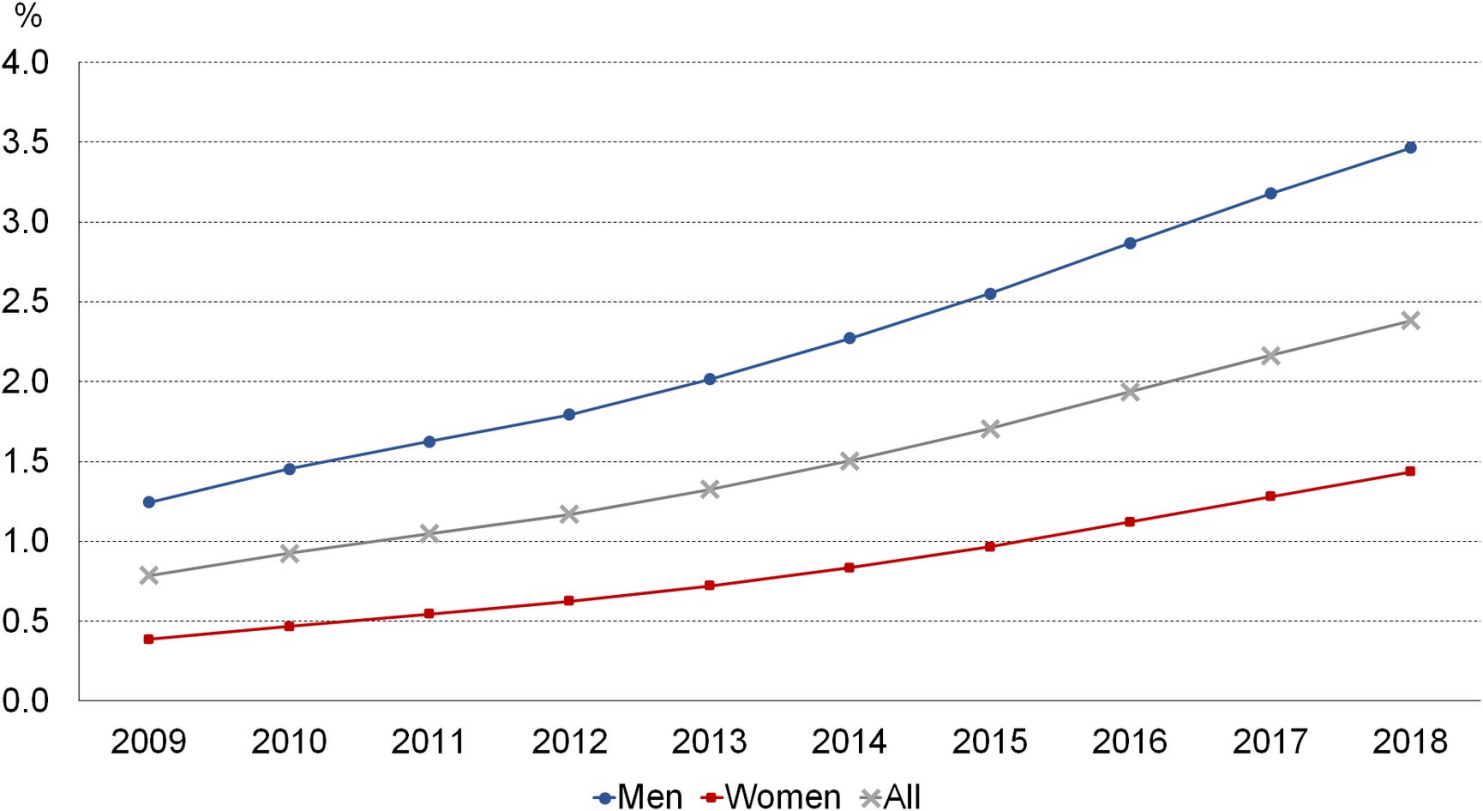
Time trends in the treatment with continuous positive airway pressure in people aged ≥20 years, age-standardized rates, France, 2009–2018.

Between 2009 and 2018, in adults aged 20 years or older, the standardized prevalence of CPAP treatment tripled (+16.0% per year on average) (Fig 4 and Table 1). The increase was statistically more marked in women and, by age group, in the youngest and oldest groups.

**Table 1:** Time trends in the prevalence of treatment with continuous positive airway pressure by gender and age group, France, 2008–2019.

	**Average annual change**
**Gender** Men Women	+14.1% +17.8%
**Age (years)** 20–39 40–59 60–79 ≥80	+17.2% +13.4% +13.6% +21.9%

### Incidence of CPAP treatment

A total of 213,635 CPAP-treated patients were newly treated, leading to an annual incidence of 4.2 per 1,000. Between 2010 and 2018, the standardized annual incidence of CPAP treatment increased 1.9-fold, with an average annual increase of 11.4% (+10.0% in men and +13.9% in women).

### Discontinuation of CPAP treatment

Among the 1,121,260 patients aged 20 years or older treated with CPAP in 2017 who did not die in 2017 or 2018, 77,040 (6.9%) stopped treatment in 2018. Only 2,140 (2.8%) of these 77,040 patients were managed with a MAS in 2017 or 2018. The proportion of patients who stopped CPAP was higher among women (8.4%) than among men (6.2%). In terms of age group, this figure fell gradually from 20.4% in the 20–24 age range to 5.1% in the 65–69 age range and then increased to reach 13.0% in those aged 90 years and older.

### Treatment with MAS

In 2017, 15,584 patients aged 20 years or older were reimbursed for a MAS, which corresponds to a rate of 0.3 per 1,000. Between 2009 and 2018, the standardized annual rate of MAS reimbursement increased 7.6-fold, representing an average annual increase of 19.7% (+17.5% in men and +23.1% in women).

## Discussion

Our study shows a high prevalence of treatment for OSA in France. In 2017, 2.3% of adults aged 20 years or older were treated with CPAP, rising to 5% among those aged 70–74 years. Moreover, the number of treated patients has risen considerably over the last decade. Between 2009 and 2018, the prevalence of CPAP treatment tripled, while the rate of patients reimbursed for a MAS (first prescription or renewal) increased almost 8-fold over the same period.

To our knowledge, this is the first study to estimate the prevalence of treatment for moderate to severe OSA in the French adult population based on exhaustive data. However, due to the underdiagnosis of OSA, our findings represent only part of the disease burden. In Canada, health administrative data were poorly accurate in identifying OSA patients [8]. For the algorithm based on PSG followed by receipt of a CPAP device, although the specificity was high (98%), the sensitivity was low (19%). In a study conducted in the French general population in 2008, only 15% of individuals with symptoms suggestive of OSA reported undergoing a sleep study [9]. A similar figure (19%) was reported in the USA in 2007 based on a questionnaire on symptoms suggestive of OSA and the electronic medical records of respondents [10]. In France, there are no estimates for the prevalence of OSA in the general population. In the international literature, prevalence surveys are scarce, with varying results depending on the definition of OSA. A literature review published in 2013 showed that prevalence ranged from 9% to 17% based on the AHI threshold of at least five apneas or hypopneas per hour [11]. With a threshold of 15, prevalence was 6%. In Europe, the largest prevalence survey was carried out in Switzerland in around 2,000 individuals, observing a much higher prevalence of moderate to severe OSA: 49.7% in men and 23.4% in women [12]. A very high prevalence of moderate to severe OSA was also found in two other studies: 14.2% of men and 7.0% of women in Spain and 24.8% of men and 9.6% of women in Brazil [13, 14]. These results cannot be extrapolated to the French population given the different study populations, notably in terms of BMI, which is a major risk factor for OSA. However, they raise questions about the scale of the disease in France. Our rate of CPAP-treated patients (2.3% in 2017) is far below these OSA prevalence estimates. From the disease to the treatment, there is a long way, which may explain part of this low rate. We may also hypothesize that OSA remains undiagnosed, because patients and health professionals minimize snoring or are unaware of excessive daytime sleepiness, which is often confused with fatigue. It is also possible that sleep examinations are not readily available in France, as sleep experts and centers are not common. In the last decade, under the authority of the French Sleep Research and Medicine Society, more than 1,000 doctors (about 100 per year) graduated in Sleep Medicine, which was recognized as a medical subspecialty in 2017.

The second important issue of our study is the marked increase in the rates of prevalence and incidence of CPAP treatment (+16% and +11% per year respectively). The rate of patients covered for a MAS also increased significantly (+20% per year). These trends took into account changes in the age structure of the French population. Changes in obesity are unlikely to explain all of the increase in CPAP treatment observed in our study. Following the sharp rise in the prevalence of self-reported obesity in adults between 1997 and 2000, this rise then slowed significantly, and between 2009 and 2012, a non-statistically significant increase of 3% was observed [15]. Surveys conducted in 2006 and 2015 based on anthropometric data did not observe any significant change in the prevalence of obesity in adults [16]. The most recent data on diabetes show a fall in the incidence of this disease between 2012 and 2017 (–2.6% per year) [17]. One explanation for the sharp increase in CPAP incidence might be the better recognition and management of OSA in France, which is in keeping with data from North America that show a 15-fold increase in OSA diagnoses between 1993 and 2010 [18]. In France, efforts have been made over the last decades to raise awareness about sleep disorders among general practitioners and the general population.

The prevalence of CPAP treatment was higher in men, which is consistent with the literature showing a prevalence of OSA that is two to three times higher in men than in women [19, 20]. In our study, in both men and women, the prevalence of CPAP treatment was the highest in the 70–74 year age group. Studies show that the prevalence of moderate to severe OSA increases with age up to 55–60 years, but this rise is followed by a plateau if OSA is defined by AHI alone and by a decrease if sleepiness is incorporated [21, 22]. These studies also show a higher severity of OSA in the youngest age group, suggesting that the lower prevalence in the oldest group could relate to a “survivor” effect, with severe OSA patients dying prematurely.

The increase in the prevalence of CPAP treatment was more marked in women. This may reflect the differential evolution of OSA risk factors by sex, such as that observed for obesity [16]. Prevalence trends in CPAP treatment may also reflect the greater reduction of underdiagnosis of OSA in women. Comparing the sex ratio of patients managed for OSA in specialized centers with those identified in general population surveys, studies suggest that OSA might be diagnosed less frequently in women [23, 24].

We found significant regional variations in the prevalence of CPAP treatment, which are consistent with those observed for the prevalence of obesity [15, 25]. Nonetheless, regional disparities in OSA diagnosis and management cannot be excluded.

A recent systematic review pointed to low socioeconomic status as a risk factor for OSA [26]. In line with this finding, we observed a greater prevalence of CPAP treatment in areas with a higher deprivation index, which probably reflects the higher prevalence of obesity in more disadvantaged areas [27, 28]. Nonetheless, we did not find any difference in CPAP prevalence according to entitlement to the CMU-C, which is free supplementary health insurance based on income. By contrast, the prevalence of diabetes was found to be twice as high in CMU-C beneficiaries than in other people [29]. These results may suggest the lower diagnosis and/or treatment of OSA in the most socioeconomically disadvantaged people. A lower adherence to CPAP treatment in socioeconomically disadvantaged patients could also play a role [30].

Regarding comorbidities, we analyzed specific chronic diseases in patients treated for OSA through the frequency of individuals who were exempt from a co-payment due to a long-term condition (ALD). This analysis focused on beneficiaries of the general health insurance scheme, irrespective of age, so that the results could be compared with published data [31]. In 2017, the proportion of patients treated with CPAP and categorized under ALD due to a post-stroke disability, heart disease, or diabetes were between 3 and 5 times higher than those seen for all beneficiaries of the general scheme. The relationship between OSA and diabetes is well known. OSA is associated with changes in glucose metabolism and is a risk factor for type 2 diabetes, whereas diabetes is a risk factor for sleep-related respiratory disorders [32]. Regarding cardiovascular diseases, OSA causes hypertension and is associated with a higher incidence of stroke, heart failure, heart rhythm disorders, and coronary heart disease [33]. However, these comorbidities may also complicate CPAP adherence [30].

In our study, 7% of patients treated with CPAP in 2017 were not reimbursed in the following year, and among them, only 2.8% were managed with a MAS in 2017 or 2018. These results are difficult to interpret. Some patients may have recovered (weight loss, surgical treatment). Among patients who continued treatment, some of them do not correctly use the CPAP. A systematic review from 2016 estimated overall treatment nonadherence to be 34.1%, with no significant improvement over the past 20 years [34].

The main strength of our study is its use of an exhaustive database covering all healthcare reimbursements for the entire French population. Our study has several limitations. Due to the substantial underdiagnosis of OSA, health insurance reimbursement data only gives a partial view of the OSA burden in France. Moreover, our data do not consider other OSA treatments (i.e., other types of ventilation, positional therapy) or OSA cases that are resolved following surgical treatment or weight loss, while they include treated patients who do not comply with treatment. Another limitation is the lack of information about the reasons for CPAP treatment discontinuation.

In conclusion, our study shows a sharp increase in treatment for OSA in France. The prevalence of CPAP treatment tripled between 2009 and 2018, while the incidence increased 1.9-fold between 2010 and 2018. The marked increase in the incidence of CPAP treatment may reflect the better recognition of OSA in France. However, the prevalence of CPAP treatment remains below the expected prevalence of moderate to severe OSA according to international data. Further studies should identify the obstacles to the optimal management of patients with OSA and the factors determining treatment failure.
